# The Potential of *Thymus zygis* L. (Thyme) Essential Oil Coating in Preventing Vulvovaginal Candidiasis on Intrauterine Device (IUD) Strings

**DOI:** 10.3390/pharmaceutics17101304

**Published:** 2025-10-07

**Authors:** Gulcan Sahal, Hanife Guler Donmez, Herman J. Woerdenbag, Abbas Taner, Mehmet Sinan Beksac

**Affiliations:** 1 Biotechnology Division, Department of Biology, Faculty of Sciences, Hacettepe University, Beytepe, Ankara 06800, Türkiye; 2 General Biology Division, Department of Biology, Faculty of Sciences, Hacettepe University, Beytepe, Ankara 06800, Türkiye; hnftnr@gmail.com; 3 Department of Pharmaceutical Technology and Biopharmacy, Groningen Research Institute of Pharmacy (GRIP), University of Groningen, Antonius Deusinglaan 1, 9713 AV Groningen, The Netherlands; h.j.woerdenbag@rug.nl; 4 Department of Medical Microbiology, Faculty of Medicine, Yuksek Ihtisas University, Çankaya, Ankara 06530, Türkiye; abbastaner@gmail.com; 5 Department of Obstetrics and Gynecology, Faculty of Medicine, Hacettepe University, Ankara 06100, Türkiye; beksac@hacettepe.edu.tr; 6 Department of Obstetrics and Gynecology, Liv Hospital Ankara, Istinye University, Istanbul 34408, Türkiye

**Keywords:** antifungal effect, biofilm formation, *Candida*, intrauterine device (IUD), *Thymus zygis* L. essential oil, vulvovaginal candidiasis

## Abstract

**Background/Objectives:** Fungal colonization and biofilm formation on intrauterine device (IUD) strings are known to contribute to recurrent infections and decreased contraceptive efficacy. This study aims to develop a novel approach to prevent *Candida* reservoir and biofilm formation on IUD strings, thereby lowering the risk of IUD-associated vulvovaginal candidiasis (VVC). **Methods:** Cervicovaginal samples were collected from human cervix using a sterile cytobrush, avoiding microbial contamination. Cytological examination using the Papanicolaou method was performed to detect the presence of *Candida*. The antifungal effect of the essential oils (EOs) was determined by broth dilution and disk diffusion methods. Antifungal and biofilm inhibitory effects of *Thymus zygis* (Tz) EO-coated IUD strings were determined by agar diffusion and crystal violet binding assays, while fungal growth on the coated strings was assessed using Scanning Electron Microscopy (SEM) and Energy-Dispersive X-ray (EDX) analysis. **Results:** Tz EO exhibited significantly lower minimum inhibitory concentration (MIC ≤ 0.06 µL/mL) and minimum fungicidal concentration (MFC = 0.24 µL/mL) values compared to *Melaleuca alternifolia* (Ma) EO (MIC > 0.24 µL/mL, MFC = 1.95 µL/mL), along with larger zones of inhibition (ZOI) against both *Candida albicans* (110.0 ± 6.0 mm vs. 91.3 ± 7.0 mm) and *Candida glabrata* (84.0 ± 13.1 mm vs. 50.0 ± 9.2 mm), indicating a stronger antifungal potential. On IUD strings coated with 4% (40 μL/g) Tz EO in hypromellose ointment, the biofilm formation of both *C. albicans* and *C. glabrata* strains was inhibited by 58.9% and 66.7%, respectively, as confirmed by SEM and EDX. **Conclusions:** Tz EO-coated IUD strings effectively inhibit *Candida* growth, suggesting a promising natural strategy to reduce recurrent IUD-associated fungal infections. However, before these results can be translated to clinical practice, additional research is needed. Future investigations may encompass an extended number of *Candida* isolates, stability and release studies of the EO in relation to the formulation, toxicity to vaginal mucosa, epithelial cells and sperm motility, and the effect on vaginal microbiotia.

## 1. Introduction

Vulvovaginal candidiasis (VVC), caused by *Candida* species, is a common gynecological condition, with an estimated 75% of women experiencing it at least once in their lifetime [1,2]. Several factors, including hormonal changes, antibiotic use, uncontrolled diabetes, weakened immunity, poor personal hygiene, and intrauterine device (IUD) use, may increase the risk of VVC [3,4]. Treatment typically involves topical azoles such as clotrimazole or miconazole, or a single oral dose of fluconazole [5]. In cases of azole resistance, vaginally applied boric acid is considered an effective alternative [6].

The ability of *Candida* species to form biofilms is a key factor in recurrent infections, affecting 40–50% of women [7]. Biofilm formation enables these fungi to colonize and persist on biotic and abiotic surfaces as complex, three-dimensional structures embedded in a self-produced extracellular matrix [8]. Such biofilms can be up to 1000-fold more resistant to antifungal agents than their planktonic counterparts [9]. Among *Candida* species, *C. albicans* biofilms exhibit particularly high antifungal tolerance, showing 5- to 8-fold greater resistance to fluconazole and other azoles compared with planktonic cells. The resistance is mainly attributed to the protective extracellular matrix, altered gene expression, and the presence of persister cells within the biofilm *Candida* forms [10,11].

**Figure 1:**
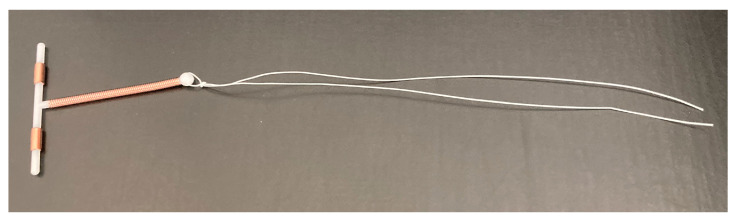
Image of the model Copper T 380A intrauterine device (IUD) employed in coating experiments.

IUDs are widely used as effective, safe, and long-term contraceptives [12]. They consist of a T-shaped body with a string at the end (see Figure 1). Their contraceptive action is based on the release of copper ions or levonorgestrel after placement into the uterus. The string extends into the vaginal canal and is used to check IUD placement and facilitate removal [13,14]. However, IUD use has been associated with an increased risk of VVC by enhancing virulence factors [15,16,17]. *Candida* species can adhere to all parts of IUDs, including both the wire and the string [4].

Essential oils (EOs), produced and stored in various plant parts, have long been used for medicinal purposes. Most are obtained by hydrodistillation and recovered via a water-cooled condenser [18,19]. They are valued for their diverse biological activities, particularly antimicrobial effects [20,21,22]. Notably, EOs such as from *Cinnamomum verum* (cinnamon), *Cymbopogon martinii* (palmarosa), *Cymbopogon citratus* (lemongrass), *Syzygium aromaticum* (clove), *Origanum vulgare* (oregano), and *Melaleuca alternifolia* (tea tree) have shown strong antifungal activity, with significant inhibitory effects against various *Candida* species and other opportunistic fungi [23,24]. These properties highlight their potential as coating agents for biomaterials.

*Thymus zygis* (Spanish thyme; Tz) essential oil (EO) has been reported to exhibit antifungal and biofilm-inhibitory effects, particularly against *Candida* species [25,26]. Its high content of phenolic compounds such as thymol and carvacrol has been closely linked to its antimicrobial efficacy [27]. These findings suggest that Tz EO may be a promising natural alternative for managing *Candida*-related infections and biofilm-associated complications [28].

Therefore, the aim of this study was to evaluate the inhibitory effects of Tz EO against clinical *C. albicans* V6 and *C. glabrata* V23 strains. *Melaleuca alternifolia* (Ma) EO was used as a reference EO to assess the antifungal potential of Tz EO under the same experimental conditions, since its activity against *Candida* species has been well documented in previous studies [21,29,30]. We further aimed to establish a novel approach to prevent *Candida* colonization and biofilm formation on IUD strings. Coating IUD strings with Tz EO in a hypromellose ointment base (Tz EO-coated IUD strings) may offer a potential strategy to reduce the risk of VVC-associated IUD infections. This approach could help decrease recurrent infections and enhance the overall effectiveness and safety of IUDs.

## 2. Materials and Methods

### 2.1. Essential Oils

Essential oil (EO) from *Thymus zygis* L. (Tz; Spanish thyme) (Lamiaceae) and EO from *Melaleuca alternifolia* Maiden & Betche (Cheel) (Ma; tea tree) (Myrtaceae) were purchased from Tisserand Aromatherapy (West Sussex, UK). Both oils had been obtained by hydrodistillation of fresh plant material.

### 2.2. Determination of EO Components by Gas Chromatography-Mass Spectrometry (GC-MS) Analysis

The composition of Ma EO was analyzed by gas chromatography–mass spectrometry (GC–MS) using an Agilent 7890B GC/5977A Series MSD System (Agilent Technologies, Santa Clara, CA, USA) at HUNITEK (Hacettepe University Advanced Technologies Application and Research Center, Ankara, Turkey). The composition of Tz EO was determined in an earlier study [31]. Components were identified by comparing the mass spectra with library data (NIST14.L, minimum quality: 80; W10N14.L, minimum quality: 20).

### 2.3. Cervicovaginal Sample Collection and Cytological Evaluation

Cervicovaginal samples were collected at the Gynecology Department of Hacettepe University Hospital between June 2019 and June 2020. After speculum insertion, cervical discharge was obtained with a sterile cytobrush and fixed immediately with 96% ethanol. The samples were processed using the Papanicolaou (Pap) method, as described by Donmez et al. [32]. Briefly, fixed slides were sequentially washed with decreasing concentrations of alcohol, rinsed with distilled water, and stained with Harris’ hematoxylin for 2 min, followed by differentiation in 1% hydrochloric acid–alcohol and rinsing with distilled water. Slides were then stained with Orange G and EA 65 for 3 min, rinsed with 95% ethanol, and mounted with Entellan (Merck KGaA, Darmstadt, Germany). Cytological examination was performed with a camera-equipped light microscope (Leica Microsystems, Wetzlar, Germany, 4000B). A smear sample containing fungal cells was selected for isolation and identification. All samples were evaluated blindly by one author (H.G.D.) according to the Bethesda criteria [33].

### 2.4. Microbial Growth of Cervicovaginal Samples

Our initial aim was to grow mixed cultures, as the microbial composition of cervicovaginal samples, including bacteria, can influence the growth of yeasts such as *Candida*. For this purpose, the cytobrush was immersed in 10 mL Brain Heart Infusion (BHI) broth (pH 7; Lab M Ltd., Heywood, Lancashire, UK), a nutrient-rich medium supporting both bacterial and fungal growth. Mixed cultures were incubated at 37 °C for 72 h to mimic human body conditions. The streak plate method [34] was used to isolate individual colonies. Colonies suspected to be yeasts were stained and examined under a light microscope (Leica DM 4000B), and one colony containing yeast cells was selected for further isolation. The isolate was cultured in BHI broth (pH 7) supplemented with 10% glycerol and stored at −20 °C for subsequent identification and experiments.

### 2.5. Microorganisms

Two *Candida* strains were used: one was isolated from a cervicovaginal sample after cytological evaluation and identification, and the other was a *C. albicans* V6 strain previously isolated from the vagina in one of our earlier studies [35]. Both strains were selected for their clinical relevance and biofilm-forming capacity, a key factor in pathogenicity and resistance to treatment.

### 2.6. Microbial Identification of the Cervicovaginal Isolate and Evolutionary Analysis

Evolutionary analyses were performed in MEGA X, version 11.0.13 (Pennsylvania State University, University Park, PA, USA) [36] using the Maximum Likelihood method with the Tamura–Nei model [37]. The isolated fungal strain was inoculated into BHI agar (pH 7) and incubated at 37 °C for 48 h. Identification was achieved by 18S ribosomal RNA gene sequence analysis (BM Labosis, Ankara, Turkey, 2020). DNA was extracted using the EurX GeneMATRIX Plant & Fungi DNA Isolation Kit (Gdańsk, Poland), and DNA concentration and purity were assessed spectrophotometrically with a Nanodrop 2000 (Thermo Scientific, Wilmington, DE, USA).

For PCR amplification, universal primers ITS1 (5′-TCCGTAGGTGAACCTGCGG-3′) and ITS4 (5′-TCCTCCGCTTATTGATATGC-3′) were used. The PCR protocol included initial denaturation at 95 °C for 5 min, followed by 40 cycles of 95 °C for 45 s, 57 °C for 45 s, and 72 °C for 60 s, with a final extension at 72 °C for 5 min and hold at 4 °C. Reactions were performed in a Kyratec thermocycler (Kyratec, Mansfield, QLD, Australia). PCR products were resolved by electrophoresis on 1.5% agarose gels prepared with 1× TAE buffer, run at 100 V for 90 min, and visualized under UV light after ethidium bromide staining.

Single-step PCR amplification yielded fragments of ~1470 and ~700 bp. PCR was carried out using FIREPol^®^ DNA Polymerase (Solis Biodyne, Tartu, Estonia). Single bands were obtained for all seven samples, which were subsequently purified with the HighPrep™ PCR Clean-up System (MAGBIO Genomics, Gaithersburg, MD, USA).

For Sanger sequencing, samples were analyzed at Macrogen (Macrogen Europe B.V., Amsterdam, The Netherlands) using the ABI 3730XL platform and the BigDye Terminator v3.1 Cycle Sequencing Kit (Applied Biosystems, Foster City, CA, USA). Reads generated with the ITS1–ITS4 primers were assembled into consensus sequences using the CAP contig assembly algorithm in BioEdit software, version 7.2 (Tom Hall, Ibis Biosciences, Carlsbad, CA, USA) [38]. The 18S rRNA gene sequence was deposited in GenBank under accession number OR648269 (https://www.ncbi.nlm.nih.gov/nuccore/OR648269, accessed on 28 July 2025).

### 2.7. Microbial Growth Conditions and Harvesting

Microbial growth and harvesting were performed as described by Sahal et al. [39,40]. Briefly, the two *Candida* strains were first grown in 10 mL BHI broth, (which is also known to support robust biofilm formation) [41], at 37 °C for 48 h to obtain precultures. Subsequently, 1.5 mL of precultures were inoculated into 30 mL BHI broth and incubated at 37 °C for 24 h to obtain main cultures. Main cultures were harvested by centrifugation three times at 3220× *g* for 10 min at 5 °C (Eppendorf 5810R, rotor A-4-62, Hamburg, Germany). Microorganisms were washed with 10 mM potassium phosphate buffer (pH 7) and adjusted to a 2.0 McFarland standard to prepare the *Candida* test suspensions. These growth conditions were selected to mimic uterine conditions, thereby providing a relevant environment for evaluating the efficacy of treatments against clinical isolates.

### 2.8. Determination of Minimum Inhibitory Concentration (MIC), Minimum Fungicidal Concentration (MFC) and Zone of Inhibition (ZOI) of Tz and Ma EOs

Two-fold dilutions of Tz and Ma EOs were prepared in 96-well plates over a concentration range of 0.06–500 μL/mL in BHI broth [42]. During serial dilution, the EOs were mixed with the medium by pipetting equally in each well to ensure proper emulsification and dilution. Following this, *Candida* test suspensions (10 μL, adjusted to a 2.0 McFarland standard) were inoculated into 100 μL of BHI broth containing the diluted EOs. Plates were incubated at 37 °C for 48 h, after which MIC values were determined visually and recorded. Following MIC determination, 10 μL from wells without visible growth were inoculated onto BHI agar plates and incubated at 37 °C for 48 h. The lowest concentration with no colony growth was recorded as the MFC value.

Zones of inhibition were determined using the Kirby–Bauer disk diffusion method [43]. Sterile blank disks (Whatman^®^ Antibiotic Assay Discs; Cytiva, Maidstone, UK) were loaded with 20 μL of Tz or Ma EO, following published protocols [44,45] and placed onto BHI agar plates spread with 100 μL of a *Candida* test suspension (2.0 McFarland standard). Plates were incubated at 37 °C for 48 h. After incubation, inhibition zones were measured as the radius (r) (mm) from the disk center to the edge of the clear zone and expressed as diameters (2r). All experiments were performed in triplicate. MIC and MFC values are reported as medians, and ZOI diameters as means.

### 2.9. Preparation and Characterization of Tz EO-Coated IUD Strings

The string of a model Copper T 380A IUD (Figure 1) was coated with Tz EO using the method previously described by Sahal et al. [39]. Briefly, a hypromellose ointment base consisting of 20% (*w*/*w*) hypromellose (hydroxypropylmethyl cellulose; viscosity 400 mPa·s) dispersed in 80% (*w*/*w*) white soft paraffin was used. This formulation was prepared according to the Dutch pharmacists’ formulary *Formularium der Nederlandse Apothekers* (FNA) [46] and obtained from Fagron (Fagron, Capelle aan den IJssel, The Netherlands). The ointment is commonly used in oral pastes due to its strong adhesion to moist mucosa [47].

The hypromellose ointment was used as a vehicle and mixed with Tz EO (10, 20, and 40 μL per 0.5 g of ointment, corresponding to 2%, 4%, and 8% concentrations, respectively) under sterile conditions, yielding final concentrations of 0% (0 μL/g), 2% (20 μL/g), 4% (40 μL/g), and 8% (80 μL/g). Since the EO was trapped within the hypromellose matrix, its volatility was reduced and a more homogeneous distribution was achieved. In addition, all ointment–Tz EO mixtures were freshly prepared before each experiment to preserve activity and were carefully mixed with a sterile glass rod until a uniform consistency was obtained. IUD strings were cut into 5 mm segments and sterilized with 70% ethanol. To coat the strings, a thin layer of ointment–Tz EO mixture was spread on a sterile Petri dish, and each string segment was submerged into the mixture to ensure uniform adhesion of the coating. The coated segments were then transferred to sterile 24-well plates for biofilm formation experiments. Both uncoated and coated string segments were examined by SEM to confirm surface coverage, and coating thickness was quantified using ImageJ software, version 1.54 (National Institutes of Health, Bethesda, MD, USA).

### 2.10. Antifungal Effect of Tz EO-Coated IUD Strings

The antimicrobial activity of IUD strings coated with different concentrations of Tz EO was evaluated using agar diffusion assays. Strings coated with 0% (0 μL/g), 2% (20 μL/g), 4% (40 μL/g), or 8% (80 μL/g) Tz EO were placed on BHI agar plates previously inoculated with 100 μL of a *Candida* test suspension. Plates were incubated at 37 °C for 48 h, and inhibition zones were measured in millimeters.

### 2.11. Biofilm Inhibitory Effect of Tz EO-Coated IUD Strings

Based on the antifungal activity results, the potential biofilm inhibitory effect of IUD strings coated with 4% (40 μL/g; 20 μL Tz EO mixed with 0.5 g hypromellose ointment) was investigated. Coated strings were placed in the wells of 24-well plates containing 500 μL of BHI broth, followed by inoculation with 50 μL of a *Candida* test suspension (2.0 McFarland standard). Plates were incubated at 37 °C for 48 h, after which biofilm formation on the IUD strings was assessed by crystal violet staining, as described by Sahal et al. [39]. This method provides reliable results when materials of equal size are compared. Since the coating increased the thickness of IUD strings by more than threefold, strings coated with hypromellose ointment without EO (0% Tz EO) were used as the control rather than uncoated strings. Biofilm formation on the 0% control strings was defined as 100%, and the percentage decrease in biofilm formation was calculated using Equation (1):

% Decrease = [(Absorbance Control (560 nm) − Absorbance Treatment (560 nm))/Absorbance Control (560 nm)] × 100%(1)

### 2.12. Scanning Electron Microscopy (SEM) and Energy-Dispersive X-Ray (EDX) Analysis

Biofilm formation on uncoated IUD strings and on strings coated with hypromellose ointment without EO (0% Tz EO) and 4% (40 μL/g) Tz EO in hypromellose ointment was evaluated by scanning electron microscopy (SEM) and energy-dispersive X-ray (EDX) analysis. After incubation, images of *Candida* growth on IUD strings were obtained with a SEM (Sigma 300, Carl Zeiss Microscopy GmbH, Jena, Germany). EDX analysis was performed using a spectrometer attached to the SEM. For sample preparation, IUD strings were mounted on aluminum stubs with double-sided adhesive tape and sputter-coated with gold to improve conductivity prior to imaging.

### 2.13. Statistical Analysis

The normality of the data was assessed using the Shapiro–Wilk test. For datasets following a normal distribution, parametric tests were applied. Specifically, the independent *t*-test was used for comparisons between two independent groups, including ZOI and biofilm inhibition data. For datasets not following a normal distribution, non-parametric tests were applied; in particular MIC and MFC values, which were reported as medians, were analyzed using the Mann–Whitney U test. Statistical significance was set at *p* < 0.05. All analyses were performed using SPSS software, version 23 (IBM Corp., New York, NY, USA).

### 2.14. Sequencing Data

The yeast isolated from the single colony was identified by 18S ribosomal RNA gene sequence analysis and deposited in GenBank under accession number OR648269.

## 3. Results

### 3.1. Components of Ma and Tz EOs Estimated by GC–MS Analysis

In the first part of this study, the composition of Ma and Tz EOs was evaluated. According to GC–MS analysis, the major constituents of Ma EO were terpinene-4-ol (40.0%), γ-terpinene (16.7%), and 2-carene (7.4%) (Table 1). The composition of Tz EO has been reported previously [31]; in brief, it predominantly contained o-cymene (38.8%), carvacrol (22.7%), and thymol (20.7%).

### 3.2. Cytological Evaluation of Cervicovaginal Sample and Tested Candida Strains

**Figure 2:**
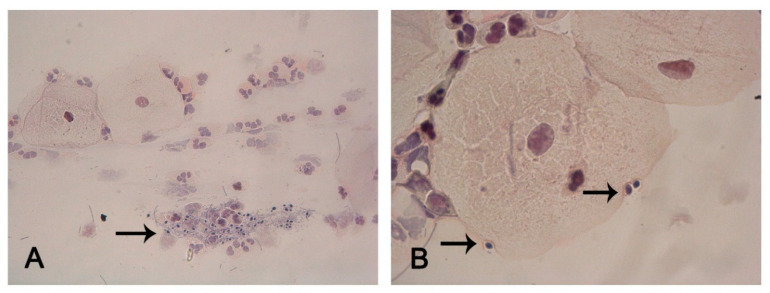
Cytological examination of cervicovaginal smear. (**A**) The group of yeast cells indicating fungal infection (arrow) (400×). (**B**) Adherence of yeast cells to the membrane of epithelial cells and formed curved invaginations on the surface of the epithelial cells (arrow), utilizing Papanicolaou staining (1000×).

Collected cervicovaginal samples were cytologically evaluated for the presence of *Candida* cells. Light microscopic examination of a sample from a woman with recurrent itching and discharge revealed yeast cells consistent with fungal infection (Figure 2). These cells were observed adhering to epithelial membranes and forming curved invaginations on their surfaces (Figure 2B). The smear sample containing *Candida* cells was isolated and identified as *Candida glabrata* by 18S rRNA analysis (GenBank accession no. OR648269; BM Labosis, Ankara, Turkey). In addition, a *C. albicans* V6 strain, previously isolated from the vagina in one of our earlier studies [35], was included in this study.

### 3.3. Antimicrobial Activity of Ma and Tz EOs

The antimicrobial activities of Ma and Tz EOs were tested against clinical *C. albicans* V6 and *C. glabrata* V23 isolates. Tz EO exhibited greater activity than Ma EO, as indicated by significantly larger ZOI diameters (*p* = 0.003 for both *C. albicans* and *C. glabrata*) (Table 2). In addition, *C. albicans* V6 was more sensitive than *C. glabrata* V23, showing significantly larger ZOI diameters (*p* = 0.010 for Tz EO; *p* < 0.001 for Ma EO). Overall, Tz EO demonstrated superior antimicrobial activity against both isolates, with *C. albicans* V6 being more susceptible to the tested EOs.

### 3.4. Antimicrobial and Biofilm Inhibitory Effect of Tz EO-Coated IUD Strings

**Figure 3:**
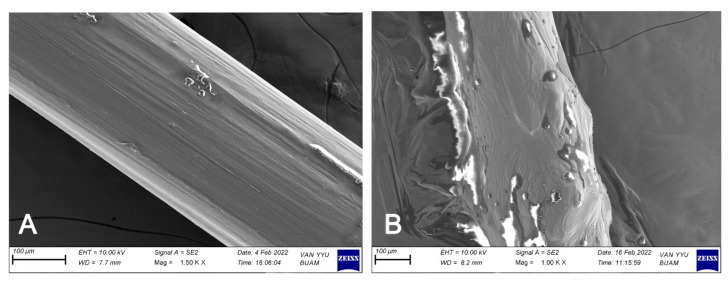
SEM images of (**A**) uncoated IUD strings (**B**) 4% Tz EO-coated IUD string at 1500× and 1000× magnifications.

IUD strings were successfully coated with hypromellose ointment, as confirmed by SEM analysis (Figure 3). The thicknesses of uncoated and coated IUD strings were 0.40 ± 0.02 mm and 1.50 ± 0.12 mm, respectively, corresponding to a coating thickness of 1.10 ± 0.11 mm.

The antifungal activity of Tz EO-coated IUD strings was evaluated by agar diffusion assays. Strings coated with 8% (*v*/*w*) Tz EO produced inhibition zones of 5.33 ± 0.58 mm against *C. albicans* V6 and 4.33 ± 1.53 mm against *C. glabrata* V23. Since inhibition was observed at this concentration, the biofilm inhibitory effect of 4% (*v*/*w*) Tz EO-coated strings was further investigated. Biofilm formation was reduced by 58.9% in *C. albicans* V6 and 66.7% in *C. glabrata* V23. No statistically significant difference was detected between the two strains (*p* = 0.470), indicating that 4% (*v*/*w*) Tz EO was similarly effective against both.

### 3.5. Inhibitory Effect of Tz EO-Coated IUD Strings by Scanning Electron Microscopy (SEM) and Energy-Dispersive X-Ray (EDX) Analysis

**Figure 4:**
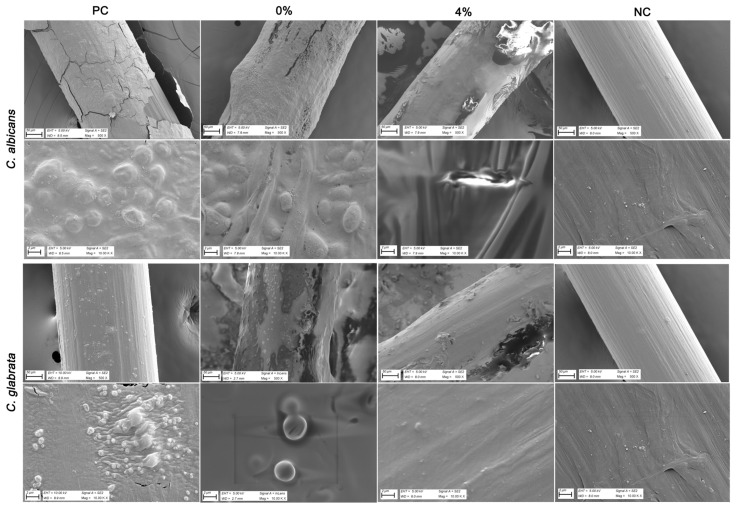
SEM images of *C. albicans* V6 and *C. glabrata* V23 biofilms formed on IUD strings under different conditions. PC: positive control (uncoated IUD strings); 0%: strings coated with hypromellose ointment only (no essential oil); 4%: strings coated with 4% Tz EO in hypromellose ointment; NC: negative control (untreated sterile IUD strings). Scale bars are shown in each panel.

In the final part of the study, SEM and EDX analyses were performed to evaluate the inhibitory effect of 4% Tz EO-coated IUD strings against *Candida* strains. SEM images revealed dense cell clusters and biofilm-like structures on the uncoated control and on strings coated with hypromellose ointment without EO (0% Tz EO) (Figure 4). In contrast, markedly reduced *Candida* growth was observed on the 4% (*v*/*w*) Tz EO-coated strings (Figure 4). These findings demonstrate that coating IUD strings with 4% (*v*/*w*) Tz EO effectively suppresses growth and biofilm formation of both clinical *Candida* isolates.

**Figure 5:**
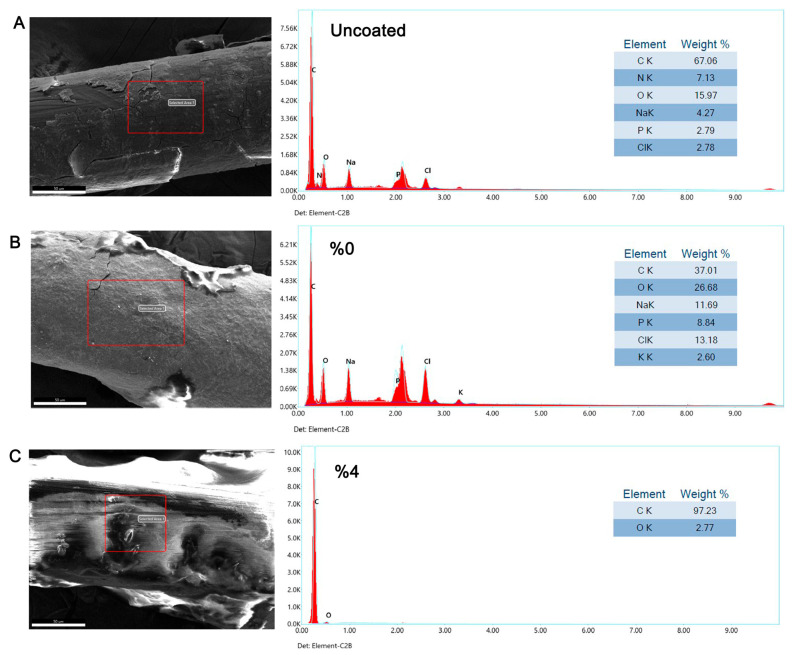
SEM and EDX analysis of (**A**) uncoated, (**B**) 0% and (**C**) 4% Tz EO-coated IUD strings after incubation with *C. albicans* V6 strain. The red box indicates the area analyzed in the figure.

**Figure 6:**
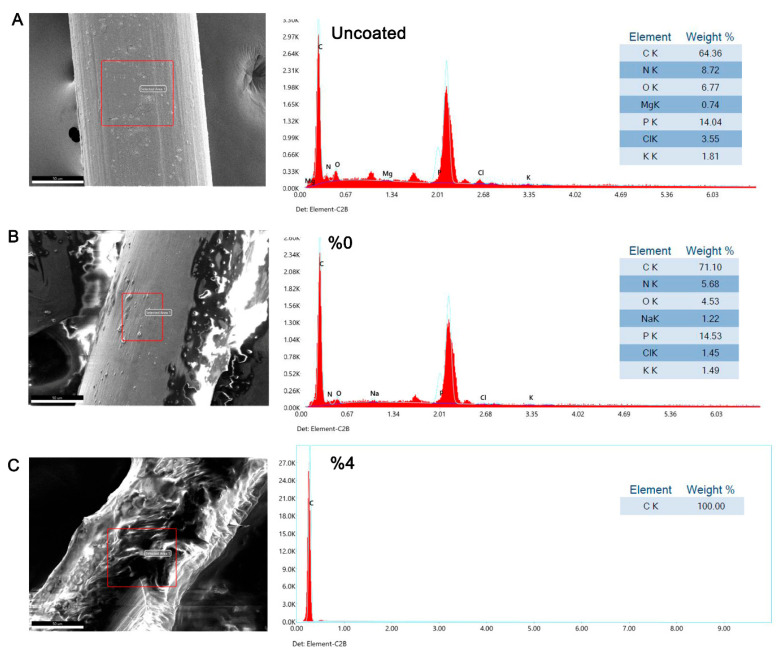
SEM and EDX analysis of (**A**) uncoated, (**B**) 0% and (**C**) 4% Tz EO-coated IUD strings after incubation with *C. glabrata* V23 strain. The red box indicates the area analyzed in the figure.

EDX analysis revealed elemental signals consistent with microbial growth on uncoated strings and on strings coated with hypromellose ointment without EO (0% Tz EO), in addition to the expected presence of carbon (Figure 5 and Figure 6). Elevated levels of oxygen (O), sodium (Na), and phosphate (P) were detected in these regions. In contrast, predominantly carbon was detected on 4% (*v*/*w*) Tz EO-coated strings after incubation with *C. albicans* V6 and *C. glabrata* V23, indicating reduced microbial deposition (Figure 5 and Figure 6).

## 4. Discussion

Vulvovaginal candidiasis (VVC) is one of the most common infections, affecting approximately 75% of women at least once in their lifetime [48]. Although more than 20% of women carry *Candida* species in their vaginal flora without symptoms [49], recurrent VVC is frequent and significantly impairs quality of life, with reduced physical function and mental health compared to women without recurrent infections [48]. In addition to predisposing factors such as hormone replacement therapy, uncontrolled diabetes mellitus, immunosuppression, and the use of antibiotics and glucocorticoids, intrauterine device (IUD) use has also been associated with an increased risk of VVC [50]. While IUDs are highly effective contraceptives, microbial biofilms on their surfaces (both wire and string) increase infection risk [4,16,17]. In particular, IUD strings have been reported to promote *Candida* colonization and biofilm formation [48,50]. Biofilm formation further complicates treatment by enhancing resilience and antifungal resistance in *Candida* species [51]. Therefore, the primary aim of this study was to investigate whether coating IUD strings with essential oils (EOs) in a hypromellose ointment base could inhibit *Candida* colonization and biofilm formation.

To identify the most effective EO, we compared the properties of Ma and Tz EOs, both long recognized for diverse biological activities and traditional medicinal use [20,21,22,52]. GC–MS analysis of Ma EO revealed terpinene-4-ol, γ-terpinene, and 2-carene as major compounds, consistent with previous reports [53,54]. Tz EO predominantly contained o-cymene, carvacrol, and thymol, in agreement with its known chemotypes characterized by phenolic dominance [25,26,27,55,56].

*Candida albicans* is the predominant cause of VVC, although *C. glabrata*, *C. tropicalis*, and *C. parapsilosis* are also implicated [57]. In this study, *C. glabrata* was isolated from a cervicovaginal sample. Clinically, however, recurrent VVC is more often associated with *C. albicans* biofilms, while *C. glabrata* infections are linked to higher treatment failure and antifungal resistance [48]. Consistently, *C. glabrata* V23 displayed greater resistance to both EOs, as reflected by significantly smaller ZOI diameters (Table 2).

Ma EO is well documented for its antibacterial and antifungal properties but has been associated with irritation and allergic reactions upon topical application [52]. In contrast, Tz EO has long been used for its antibacterial, antifungal, antioxidant, anti-inflammatory, and anti-biofilm effects [58,59,60,61]. It is generally recognized as safe (GRAS status) by the FDA (21CFR182.20), though hypersensitivity reactions have been reported [62,63,64,65]. Thyme EO has been shown to be non-cytotoxic against in vitro culture (tumor) cell lines at the following concentrations: 228.78 µg/mL for the MOLT-4 cell line (95% confidence interval: 118.23–442.66), 52.65 µg/mL for the MCF-7 cell line (95% confidence interval: 11.35–244.13), and 68.59 µg/mL for the H460 cell line (95% confidence interval: 22.49–209.09) [66]. The MICs of Tz EO (≤0.06 µL/mL) against the tested clinical *Candida* isolates calculated as ≤55 µg/mL in this study (Table 2) were lower than the concentrations required to cause cytotoxicity [66].

Beyond its safety profile, Tz EO showed greater antifungal activity than Ma EO, as evidenced by lower MIC/MFC values and significantly larger ZOI diameters (Table 2). This superior activity may be attributed to its major components thymol and carvacrol, which disrupt fungal membrane integrity and interfere with ergosterol biosynthesis, leading to cell death and biofilm inhibition [27,67]. Therefore, Tz EO was selected for coating experiments in the subsequent phases of this study.

In recent years, considerable research has focused on the efficacy of biomaterials coated with antimicrobial agents for preventing biofilm formation and implant-associated infections [60]. We previously demonstrated that silicone rubber pieces coated with lemongrass EO could reduce *Candida*-associated biofilms [39]. However, the anti-*Candida* effects of EO-coated IUD strings had not been demonstrated until now. In the present study, we developed a novel IUD string coating with Tz EO, which was identified as the most effective EO. Hypromellose ointment (hydroxypropyl methylcellulose ointment) was used as an adhesive vehicle. Since EOs are usually applied in diluted form to avoid irritation [68], the hypromellose ointment base was considered suitable. This ointment (20% hypromellose in white soft paraffin) is commonly used in oromucosal pastes and adheres well to moist mucosa [46]. This property makes it a suitable material for coating biomaterials which are in contact with mucous surfaces as well (FDA; 21 CFR 172.874). Such adhesive and biocompatible properties, together with its moisture-retaining capacity, make hypromellose a suitable vehicle for coating biomaterials in contact with mucosal surfaces (FDA; 21 CFR 172.874) [69]. Additionally, hypromellose ointment has the ability to retain moisture, creating a hydrated environment at the biomaterial interface. This moisture retention capability is particularly beneficial for preventing dryness and maintaining optimal vehicle for EOs [70].

SEM analysis confirmed effective coating of IUD strings, with a thickness of 1.1 ± 0.11 mm (Figure 3). This provides a reference for future coating studies. Importantly, 4% Tz EO-coated strings inhibited biofilm formation by both *C. albicans* V6 and *C. glabrata* V23 strains for the first time (Figure 4), which was further corroborated by SEM-EDX analysis (Figure 5 and Figure 6). SEM images revealed a marked reduction in *Candida* growth on 4% Tz EO-coated strings compared with controls. In parallel, EDX results showed higher oxygen (O), sodium (Na), and phosphorus (P) levels in uncoated and in strings coated with hypromellose ointment without EO (0% Tz EO) consistent with microbial deposition, whereas 4% Tz EO-coated strings predominantly showed carbon (Figure 5 and Figure 6). Similar mineralization patterns have been associated with biofilm formation on other biomaterials, including urinary stents [71]. In a previous study focusing on biofilm formation by *Enterococcus faecalis* on root canal wall of teeth an increase in the atomic percentages of calcium and phosphorus was shown using EDX analysis [72]. The composition of biofilms varies depending on the type of microorganisms and the surfaces which they grow on, but there is always a relationship between increased mineralization and the formation of biofilms [73].

It should be noted that the release and protective effect of Tz EO may be limited, as IUDs are intended for long-term use. Under our experimental conditions, the hypromellose-based coating may dissipate within a few days. Nevertheless, this study provides the first evidence that Tz EO-coated IUD strings can inhibit *Candida* growth and biofilm formation. Some studies explore release kinetics and long-term EO activity on different matrices [74]. Future studies involving controlled release systems, such as microencapsulation [75], may prolong EO release and enhance the durability and safety of coated IUD strings. These modifications may lead to longer-lasting and safer protection for individuals using IUDs.

Another crucial point to consider is whether the Tz EO coating on IUD strings may affect the contraceptive properties of IUDs. An IUD functions by preventing fertilization. The copper-IUD releases copper ions into the uterine cavity, creating a toxic microenvironment for sperm and embryos. The hormonal IUD releases 20 µg of levonorgestrel daily, thickening cervical mucus and inhibiting sperm motility and viability [76,77]. Additionally, both types of IUD can lead to localized foreign body reactions within the uterus [78]. In our study, the focus was on the IUD string, which extends into the vaginal canal and does not play a direct role in contraception. This raises the question of whether coating the IUD string with Tz EO could have any impact on contraception. The direct effects of Tz EO on sperm are not well-documented in the literature. However, thymol, one of the main compounds of Tz EO, has been shown to reduce fertility in male albino Wistar rats. Thymol decreased testis weight, sperm count, and motility while increasing the number of abnormal sperm in rats [79]. Similarly, thymol may reduce fertility in humans by reducing sperm count, motility, and vitality [80]. However, before any potential clinical application, studies on local toxicity, particularly on vaginal mucosa, and on the impact of the formulation on sperm motility should be conducted to ensure safety, as any suggestion that the coating could contribute to contraceptive function remains hypothetical and would require comprehensive reproductive safety evaluations before being considered.

All findings of this study provide preclinical evidence supporting the potential of Tz EO-coated IUD strings to enhance the performance of IUDs. However, limitations, including the use of only a single clinical isolate of *C. glabrata*, the absence of controlled release and stability data for the EO hypromellose formulation, the lack of cytotoxicity and local safety evaluations (such as effects on vaginal mucosa, epithelial cells and sperm motility), and the absence of an assessment of the possible impact on commensal vaginal microbiota, need to be addressed through further investigations. Addressing these aspects is needed to ensure the safety, durability, and eventual clinical applicability of this approach.

## 5. Conclusions

In conclusion, this study shows that Tz EO exhibits strong antifungal and antibiofilm activities against clinical *C. albicans* and *C. glabrata* isolates and that coating IUD strings with Tz EO in a hypromellose adhesive ointment reduces fungal colonization. The results of this study with IUD strings coated with Tz EO in hypromellose adhesive ointment may contribute to preventing recurrent VVC infections and enhancing the safety of IUDs by inhibiting candidal colonization on IUD strings. However, further strengthening of the presented preclinical findings and translation to the clinical situation requires additional experiments. They should encompass the inclusion of a larger number of *Candida* isolates and conduct comparisons with reference strains or conventional antifungal agents. Moreover, stability and long-term performance tests, controlled release assays, cytotoxicity testing, evaluation against a broader microbial spectrum, microbiome impact analyses, and sperm motility studies remain necessary to establish clinical relevance and safety. From a pharmaceutical technological viewpoint, the possibility to formulate biologically active EOs into hypromellose ointment with good attaching properties to both wet mucosa and biomaterials offer promising perspectives and fits in a personalized medicine approach.

## Figures and Tables

**Table 1:** Components of Ma EO ordered upon increasing retention time.

Components of Ma EO	Concentration
1. (−)-alpha-Thujene	1%
2. (−)-alpha-Pinene	2%
3. (+)-Sabinene	0.25%
4. (−)-beta-Pinene	0.63%
5. beta-Myrcene	0.67%
6. (+)-alpha-Phellandrene	0.32%
7. 2-Carene	7.38%
8. p-Cymene	6.54%
9. Eucalyptol	4.42%
10. D-Limonene	0.87%
11. gamma-Terpinene	16.72%
12. Sabinene hydrate	0.15%
13. Cyclohexene, 1-methyl-4-(1-methyle thylidene)-	3.29%
14. 4-Thujanol	0.41%
15. 2-p-Menthen-1-ol	0.46%
16. 2-p-Menthen-1-ol	0.33%
17. Terpinen-4-ol	39.99%
18. Terpineol	3.09%
19. trans-Piperitol	0.17%
20. (3S,4S)-Hept-1-en-6-yne-3,4-diol	0.17%
21. Endo-2-bornyl carbanilate	0.13%
22. (−)-alpha-Gurjunene	0.42%
23. Caryophyllene	0.45%
24. 10s,11s-Himachala-3(12),4-diene	1.27%
25. (4-alpha,-5-beta,-6-alpha,-7-alpha,-10-alpha)-1-Aromadendrene	0.35%
26. (E)-2-epi-beta-Caryophyllene	0.56%
27. 1-Isopropyl-4,7-dimethyl-1,2,3,4,5,6-hexahydronaphthalene	0.42%
28. gamma-Muurolene	0.28%
29. (+)-Ledene	1.54%
30. alpha-Muurolene	0.19%
31. Calamenene	0.25%
32. Cadina-1(10),4-diene	1.66%
33. Cubenene	0.20%
34. (−)-Spathulenol	0.24%
35. (−)-Globulol	0.32%
36. 1H-Cycloprop[e]azulen-4-ol, decahydro-1,1,4,7-tetramethyl-, [1aR-(1a.alpha.,4.beta.,4a.beta.,7.alpha., 7a.beta.,7b.alpha.)]-	0.12%
37. Di-epi-1,10-cubenol	0.21%
38. Farnesol	0.84%
39. 2,6,10,14,18,22-Tetracosahexaene, 2,6,10,11,15,19,23-heptamethyl-, (all-E)-	1.69%

**Table 2:** The Minimum inhibitory concentrations (MICs; μL/mL; median values, n = 3), the Minimum fungicidal concentrations (MFCs; μL/mL; median values, n = 3) and the diameter of the Zones of inhibition (ZOIs; mm; mean values, n = 3) of Ma and Tz EOs against the *C. albicans* V6 and *C. glabrata* V23 clinical isolates.

EOs	*C. albicans* V6			*C. glabrata* V23			
	MIC (μL/mL)	MFC (μL/mL)	ZOI (mm)	MIC (μL/mL)	MFC (μL/mL)	ZOI (mm)	p^+^ (*C. glabrata* V23 vs. *C. albicans* V6)
**Ma**	0.49	1.95	91.3 ± 7.0	1.95	1.95	50.0 ± 9.2	*p* < 0.001 ^a^ ***
**Tz**	≤0.06	0.24	110 ± 6.0	≤0.06	0.24	84.0 ± 13.1	*p* = 0.010 ^a^ *
**p^#^** **(Ma vs. Tz)**	0.034 * ^b^	0.046 * ^b^	*p* = 0.003 ^a^ **	0.034 * ^b^	0.037 * ^b^	*p* = 0.003 ^a^ **	

MIC: Minimum Inhibitory Concentration (median), MFC: Minimum Fungicidal Concentration (median). ZOI: Zone of Inhibition (mean ± standard deviation). p^+^ indicates comparisons between *C. albicans* V6 and *C. glabrata* V23 for ZOI values. The symbol p^#^ refers to comparisons between Ma EO and Tz EO for MIC, MFC and ZOI values. Statistical analyses were performed using the Independent *t*-test (a) and Mann–Whitney U test (b). Significance levels are indicated as follows: * *p* < 0.05, ** *p* < 0.01, *** *p* < 0.001.

## Data Availability

The original contributions presented in this study are included in the article. Further datasets generated and/or analyzed during the current study are available from the corresponding author upon reasonable request.

## Abbreviations

The following abbreviations are used in this manuscript:

BHI	Brain Heart Infusion
EDX	Energy Dispersive X-ray Spectroscopy
EO	Essential Oil
FNA	Formularium der Nederlandse Apothekers (Formulary of Dutch Pharmacists)
IUD	Intrauterine Device
Ma	Melaleuca alternifolia
MIC	Minimum Inhibitory Concentration
MFC	Minimum Fungicidal Concentration
SEM	Scanning Electron Microscopy
Tz	Thymus zygis
VVC	Vulvovaginal Candidiasis
ZOI	Zone of Inhibition
